# Effects of Exosomal Viral Components on the Tumor Microenvironment

**DOI:** 10.3390/cancers14143552

**Published:** 2022-07-21

**Authors:** Jing Li, Yan Zhang, Bing Luo

**Affiliations:** 1 Department of Pathogenic Biology, School of Basic Medicine, Qingdao University, Qingdao 266071, China; jingliqdu@163.com; 2 Department of Clinical Laboratory, Zibo Central Hospital, Zibo 255036, China

**Keywords:** exosome, tumor microenvironment, oncovirus, cancers

## Abstract

**Simple Summary:**

Oncogenic viral infection may lead to cancers, such as nasopharyngeal carcinoma, hepatocellular carcinoma, and cervical cancer. In addition to the tumor cells themselves, the tumor microenvironment also plays a decisive role in tumor evolution. Oncogenic viruses can affect the tumor microenvironment via exosomes influencing the occurrence and development of tumors by encapsulating and transporting viral components. This review focuses on the effects of virus-infected cancer exosomes on tumor microenvironment and tumor progression.

**Abstract:**

Exosomes are extracellular membrane vesicles with a diameter of 30–100 nm, produced by different eukaryotic cells that contain multitudinous lipids, nucleic acids, and proteins. They transfer membrane components and nucleic acids between cells, thereby performing an information exchange between cells. Many studies have shown that a variety of tumor-associated viruses can exert their biological functions through exosomes. The tumor microenvironment (TME) is very important in the occurrence, development, and chemoresistance of tumors. It is composed of tumor cells, fibroblasts, endothelial cells, immune cells, stromal cells, and acellular components, such as exosomes and cytokines. This review focuses on the effects of virus-related components secreted by tumor cells over the TME in several virus-associated cancers.

## 1. Introduction

Cancer, one of the major death causes worldwide, has become a serious threat to human health [1]. Because of this, we have never given up on the exploration of cancer treatment and its pathogenic mechanism. Cancer is a multifactorial disease. In addition to the tumor cells themselves, the tumor microenvironment plays a critical role in cancer, as the interaction between tumor cells and the microenvironment affects the occurrence and development of tumors [2]. On the one hand, tumor cells reprogram surrounding cells and shape the microenvironment by secreting various cytokines, chemokines, and other small molecules [3]. On the other hand, the tumor microenvironment affects tumor survival and progression by cells and small molecules contained in it, promoting tumor drug resistance, tumor proliferation, invasion and metastasis, and even maintaining cancer stem cell phenotype [4]. Among the various internal and external factors that cause cancer, one is the presence of some viruses; this review focuses on these virus-induced cancers.

Extracellular vesicles (EVs) are lipid bilayer vesicles secreted by cells to transport materials between cells and play a role in cell communication [5,6]. Substructures, such as exosomes, microvesicles, and apoptotic bodies are EVs. Due to their difficult purification and characterization, the newly revised ISEV2018 guideline classifies them based on the following: (1) physical EV characteristics, such as size or density; (2) biochemical components; (3) origin cells [7]. However, we generally refer to EVs with a diameter between 30–100 nm and a typical lipid bilayer structure [8] as “exosomes.” As a type of EVs, they play a leading role in the regulation of the tumor microenvironment and are considered a key factor affecting tumor development [9].This article focuses on the role in tumor progression of viral components in exosomes released from cancers.

## 2. Overview of Exosomes

Since exosomes were discovered more than 30 years ago, research on them has not stopped. Exosomes play an important role in cell-to-cell communication and can serve as potential biomarkers for numerous diseases [10,11]. Exosomes not only exchange information between tumor cells, but also between tumor and stromal cells. Tumor cell-derived exosomes are transferred to stromal cells to promote the tumor microenvironment. Similarly, exosomes released from stromal cells can promote tumor cell proliferation and invasion [10].

### 2.1. Exosome Formation and Composition

Exosomes were discovered in the in vitro culture of sheep reticulocytes by Johnstone et al. [12]. They are produced by macrophages, dendritic cells, lymphocytes, and tumor cells, and contain lipids, RNA (mRNA, miRNA, lncRNA, and other RNAs), DNA, proteins (including oncoproteins, tumor suppressors, and transcriptional regulators), as well as substances related to the cell of origin [13,14,15,16]. At present, exosomes have been found in various body fluids, such as blood, urine, amniotic fluid, and milk [17]. They can transfer biological information, such as membrane composition and nucleic acids, between adjacent or distant cells [18]. These cell communications can not only realize the information exchange between cells in the physiological state, but also involve the pathogenesis of diseases, such as tumors and neurodegenerative diseases [19].

**Figure 1:**
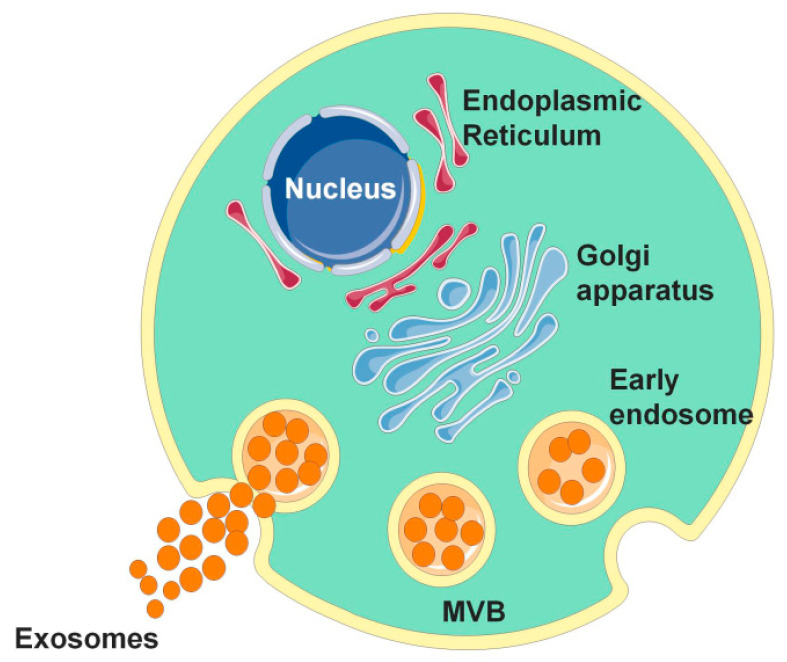
The occurrence of exosomes. The occurrence of exosomes is formed by the invagination of the cell membrane to form multivesicular bodies (MVBs). After the outer membrane of MVB fuses with the cell membrane, its contents are released into the extracellular matrix, and the secreted vesicles are called exosomes.

Unlike other EVs, exosomes are not produced directly from the plasma membrane toward an outward bud, but produced by endosomes through a complex intracellular biosynthesis process involving double invagination of the plasma membrane and the formation of multivesicular bodies (MVBs) rich in luminal vesicles (ILVs) [20]. The formation of exosomes begins with membrane internalization into early endosomes and then migration to MVB, during which proteins, mRNAs, noncoding RNAs, DNA, and some lipids are loaded. After inward budding to form ILVs, most MVBs in cells fuse with lysosomes and degrade, while others fuse with the plasma membrane, and ILVs are released extracellularly; the secreted vesicles are exosomes [21]. Exosome formation is shown in Figure 1. In exosomes, in addition to their cargo, such as proteins, lipids, and nucleic acids, ESCRT proteins and their auxiliary proteins Alix, TSG101, HSC70 and HSP90β, which regulate formation and transport of exosomes and MVB, are often referred to as “exosome marker proteins.” Simultaneously, CD9, CD63, and CD81 of the tetra-transmembrane protein family also exist in exosomes and are often used as marker proteins to identify exosomes [22].

### 2.2. Isolation and Biological Characterization of Exosomes

In research, it is very important to isolate purified exosomes. However, due to their small diameter, it is difficult to separate them. Currently, the following separation methods are commonly used: (1) supercentrifugation: the most common technique and the “gold standard” for exosome separation; requires a long time and has low purity; (2) density gradient centrifugation: can isolate exosomes with higher purity; complex operation and time consuming; (3) ultrafiltration: based on the size difference between exosomes and other substances; low cost, high enrichment efficiency, but low purity; (4) size exclusion chromatography: based on size separation; simple, fast, low cost, but not suitable for large volume samples; (5) kit extraction: relatively fast and convenient; high extraction cost, varying performance depending on manufacturer [22,23,24,25].

After successful exosome isolation, further characterization is required to determine their number and purity. Under the electron microscope, we can directly observe the morphology of purified exosomes; dynamic light scattering can detect their diameter; nanoparticle tracking analysis can not only detect their diameter, but also the concentration, and observe exosomes in real time; western blotting is a classic detection method for exosome marker protein expression; in addition, enzyme-linked immunosorbent assay can also measure the expression of exosome marker proteins; we can also detect biomarkers of exosomes by flow cytometry [23,24].

## 3. Exosomes and Viruses

Viruses are strictly intracellular parasitic non-cellular organisms, composed of a nucleic acid and protein shell, which after entering the host body, complete proliferation through adsorption, penetration, uncoating, biosynthesis, assembly, and release. After invading the host, the virus plays its pathogenic role by invading susceptible cells or damaging or changing cell function. Exosomes have been shown to play a role in viral infection; some viruses can induce biological changes by using exosome secretion pathways to transport selected products to neighboring cells [26,27].

Numerous viruses are associated with human tumors. Hepatitis B virus (HBV) and hepatitis C virus (HCV) may cause primary liver cancer [28], the human papillomavirus (HPV) is linked to cervical cancer [29], and the Epstein-Barr virus (EBV) may cause nasopharyngeal carcinoma, Hodgkin’s lymphoma, and Burkitt’s lymphoma [30,31]. These viruses exert their tumorigenic effects through genomic instability, increased cell proliferation and decreased apoptosis, and changes in DNA repair mechanisms [32].

In addition, oncoviruses can also affect tumorigenesis and development by creating a favorable tumor microenvironment [33]. Exosomes have been reported in several common oncogenic virus infections. HBV and HCV can be directly transmitted through exosomes and evade the antibody-mediated immune response through exosomes [34,35,36]. In virus-induced hepatocellular carcinoma, exosomes can affect the development of hepatocellular carcinoma through miRNA and lncRNA [37,38]. In the pathogenesis of EBV, EBV-infected B cells exert their biological functions by secreting functional viral RNA and viral proteins in exosomes [39,40,41]. In addition, exosomes can serve as cancer markers for diagnosis, staging, and treatment monitoring of virus-associated cancers [42].

## 4. Effect of Exosomal Viral Components on the Tumor Microenvironment

Among human cancers, many are closely related to the infection of pathogenic microorganisms. In 2012, about 2.2 million cancers worldwide were caused by infection, accounting for 15.4% of new cancer cases that year [43]. After 6 years, the number of cancer cases caused by infection remained high [44]. Among the infectious sources of cancer, the most important are *Helicobacter pylori*, HPV, HBV, HCV, and EBV [43,44].

As a part of tumor cells, tumor stem cells (CSCs) show the characteristics of pluripotent stem cells, which have the ability of self-renewal and infinite proliferation, and play an important role in tumor survival, proliferation, and metastasis after appropriate activation [45]. Similarly, CSCs can be infected by viruses as host cells, thus influencing changes in tumor microenvironment and tumor occurrence and development. In addition, Tu [46] proposed that, compared with differentiated and mature progenitor cells, progenitor cells with stem-like characteristics may cause persistent sequelae, autoimmune complications, and can trigger cancer after infection with oncogenic viruses, such as HBV, HPV, and EBV. For example, in EBV-associated gastric cancer (EBVaGC), Mariko Yasui et al. [47] found that EBV-encoded latent membrane protein 2A (LMP2A) helps maintain CSCs in EBVaGC, and Kong et al. [48] found the same in EBV-positive nasopharyngeal carcinoma.

Not only the tumor cells themselves, but also the interaction between the tumor microenvironment and tumor cells can have an impact on the occurrence and development of tumors. The occurrence, growth, and metastasis of tumors are closely related to the internal and external environment of tumor cells. The internal and external environment for the survival of tumor cells is called TME, which is composed of tumor cells, fibroblasts, endothelial cells, immune cells, stromal cells, and acellular components, such as exosomes and cytokines [49,50]. Evidence suggests that interactions between tumor cells and the TME may influence tumor growth and drug resistance [51]. Tumor cells can change and maintain their own survival and development conditions through autocrine and paracrine signaling and promote the growth and development of tumors. Systemic and local tissues can also limit and affect the occurrence and development of tumors through changes in metabolism, secretion, immunity, structure, and function.

### 4.1. EBV-Related Cancers

EBV is a very common gamma herpes virus that infects more than 95% of adults worldwide [52,53]. Most primary infections in childhood are asymptomatic but can cause lifelong latent infection [54]. Unlike infection in early childhood, EBV infection in adolescents and adults often causes infectious mononucleosis [55]. EBV was the first tumor-associated virus found in humans, mainly causing epithelial cell carcinoma and lymphoma [56], such as nasopharyngeal carcinoma [57], gastric cancer [58], and Burkitt lymphoma and Hodgkin’s lymphoma [59]. EBV-infected cells, such as the lymphoblastic cell line (LCL) and nasopharyngeal carcinoma (NPC) cells can release exosomes containing EBV-encoded latent membrane protein 1 (LMP1) and mature microRNAs (EBV-miRNAs), including EBV-miR-BART3 and EBV-miR-BHRF1-1 [60,61].

Hypoxia-inducible factor-1α (HIF-1α), as a transcription factor, plays a key role in cancer progression and can regulate tumor occurrence and development by promoting angiogenesis, cell proliferation, invasion, and metastasis [62]. As the main oncoprotein encoded by EBV, LMP1 plays a very important role in the carcinogenesis and tumor progression of NPC [63]. Aga et al. [64] detected HIF-1α in NPC exosomes; LMP1 could increase the HIF-1α level in exosomes. In addition, immunohistochemical analysis of exosome markers CD63 and LMP1 in NPC tissues revealed a direct correlation between them, suggesting that LMP1 could promote exosome secretion in EBV-positive tumor cells. Moreover, LMP1-positive and HIF-1α-positive exosomes could increase cell migration and invasion, affecting tumor development. LMP1 can promote the secretion of epidermal growth factor receptor (EGFR) into exosomes; exosomes containing LMP1 and EGFR are taken up by epithelial and endothelial cells and fibroblasts, activating ERK and PI3K/Akt pathways further affecting cell growth [65]. In addition, Flanagan et al. found that exosomes containing LMP1 secreted by EBV-positive cells could participate in immune regulation, inhibiting proliferation of peripheral blood monocytes and T cells in the surrounding environment, allowing immune escape of tumor cells, and playing an anti-tumor role in EBV-associated tumors, such as nasopharyngeal carcinoma and Hodgkin’s disease [66]. Klibi et al. [67] found that NPC cells can release exosomes containing galectin-9, a ligand for membrane receptor Tim-3, which can induce apoptosis of mature Th1 lymphocytes. The induction of EBV-specific CD4+ T cells by NPC exosomes can be inhibited by anti-Tim-3 and antigalectin-9 blocking antibodies. Therefore, blocking the galectin-9/Tim-3 interaction in vivo may alleviate NPC exosome-mediated Th1 inhibition and maintenance of antitumor T cell responses.

**Figure 2:**
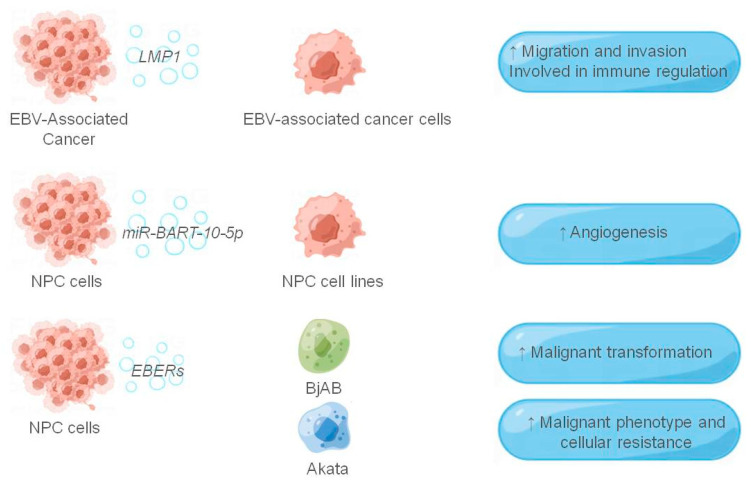
The role of exosomes in the EBV-associated tumor microenvironment. The figure shows the EBV-related components and their receptor cells that function in the exosomes, and the EBV-related components are shown in italics, and ↑ indicates that they play a role in promotion.

During latent infection, EBV-infected cells and associated tumor cells express many noncoding RNAs (ncRNAs), including EBV-encoding RNAs (EBERs), viral microRNAs, and BamHI-A Rightward Transcripts (BARTs). They can regulate viral as well as host gene expression at the post-transcriptional level, affecting viral infection and tumorigenesis [68]. Several studies have found that miRNA is an important gene regulatory factor, and its adjustment can affect the occurrence and development of cancer cells. For instance, miRNAs can be transferred to endothelial cells through EVs derived from tumor cells, affecting tumor angiogenesis [69,70]. Mature EBV miRNAs can be divided into EBV-miR-BHRF1 and EBV-miR-BART. All EBV-miR-BARTs are expressed in EBV-positive cells, while EBV-miR-BHRF1s are highly expressed in latent stage III. Wang et al. [71] found a strong correlation between the expression levels of miR-BART10-5p (a viral miRNA) and miR-18a (a member of the oncogenic miR-17-92 cluster) and NPC angiogenesis; BART10-5p and miR-18a directly co-target Spry3 to regulate VEGF and HIF1-α expression, which in turn strongly promote angiogenesis in NPC. EBER1 and 2 are two small RNAs encoded by EBV, expressed in all EBV-infected cells and the most transcribed RNAs for virus latency. Ahmed et al. [72,73] found that EBERs can be released from the nucleus in exosomes from EBV-infected cells, and can play a very important role in virus immunity. Exosomes containing EBER1 can be first phagocytosed by dendritic cells (DC) cells, triggering antiviral immunity [74]. Furthermore, Yamamoto and Komano et al. [75,76] found that EBERs could increase the growth potential of EBV-negative B lymphoma cell line BJAB, increase its malignancy, and become resistant to apoptosis. In addition, EBERs may induce malignant transformation of BJAB cells by inhibiting PKR function. Similarly, EBERs can also increase the malignant phenotype and cell resistance of the Burkitt lymphoma cell line Akata. Zhang et al. [77] found high expression of EBV-miR-BART7 and EBV-miR-BART13 in NPC patients and regular secretion into the extracellular environment. In addition, after treatment, the expression levels of these two miRNAs were significantly reduced in most patients. Therefore, EBV-miR-BART7 and EBV-miR-BART13 could be used as biomarkers to monitor the therapeutic effect and disease remission of NPC patients after treatment. In addition to EBV-encoded miRNAs, Yip et al. [78] found elevated circulating EBV DNA levels in serum and plasma samples from NPC patients, and that EBV DNA load correlated with disease stage, treatment status, relapse rate, and survival rate; thus, detection of circulating EBV DNA levels might help monitor distant metastases and response to various treatments. Below, we summarize the viral components and their roles in exosomes secreted by EBV-associated cancers (Figure 2).

### 4.2. HPV-Related Cancers

HPV is a spherical double-stranded DNA virus that can be transmitted through sexual transmission and skin-mucosal contact [79,80], and can cause squamous epithelium proliferation in the human skin mucosa. In addition, HPV is also associated with many cancers, such as cervical cancer, penile cancer, vulvar cancer, vaginal cancer, anal cancer, and oropharyngeal cancer [81,82].

Many studies have shown that HPV-positive exosomes contain HPV oncogenes E6/E7. E6 and E7 are two of the six early proteins after HPV infection, which have a carcinogenic role by avoiding growth inhibitors, promoting cell proliferation, inducing angiogenesis, activating cell invasion and migration, and inhibiting cell apoptosis [83,84]. In addition, the HPV E6/E7 oncoprotein also affects the tumor microenvironment by synthesizing and releasing pro-inflammatory cytokines and chemokines, having an impact on immune function [85]. Inoculation of HPV E7-loaded exosomes to mice, induced HPV E7 CTL production, elicited CD8+ T cell-specific immunity, and abolished tumor cell growth [86]. In head and neck cancer cells (HNC), HPV(+) exosomes containing E6/E7, P16, and survivin, which can promote DC maturation, do not inhibit the expression of antigen-processing machinery components, and play a crucial role in anti-tumor immunity, leading to better prognosis of HPV(+)HNC patients [87]. E6 and E7 can not only function through the release of exosomes, but also affect exosome secretion. Honegger et al. [88] found that, in cervical cancer HeLa cells, silencing HPV E6/E7 expression promoted exosome secretion and induced cell senescence by re-inducing p53 and stimulating its target genes TSAP6 and CHMP4C. In addition, Honegger [89], Chiantore [90], and Harden et al. [91] found that HPV E6/E7 oncoprotein expression may affect the microenvironment by regulating the number and content of miRNAs carried by exosomes and promote tumor cell proliferation.

**Figure 3:**
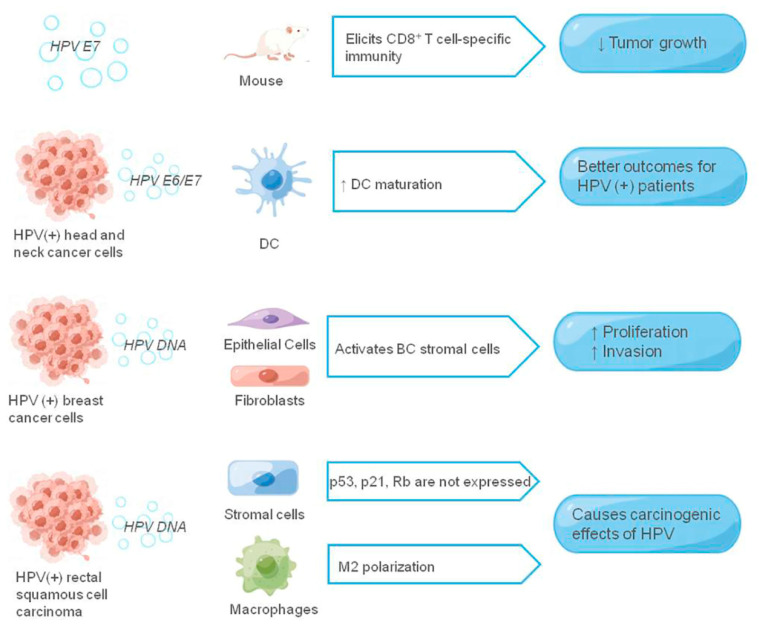
The role of exosomes in the HPV-associated tumor microenvironment. The figure shows the HPV-related components and their receptor cells that function in the exosomes, and the HPV-related components are shown in italics, ↑ means promotion, ↓ means inhibition.

HPV DNA, a novel tumor kinetic marker that can be delivered by exosomes, was found in the plasma and cervical samples of cervical cancer patients, but the role of HPV DNA in exosomes on cervical cancer transformation and progression remains unclear [92,93]. In addition to cervical cancer, exosomal HPV DNA has been linked to breast cancer (BC) and rectal squamous cell carcinoma. In breast cancer, HPV DNA can be transferred to BC stromal cells, such as fibroblasts and epithelial cells, through exosomes and activate them to promote proliferation and invasion of breast cancer cells [94]. Similarly, in rectal squamous cell carcinoma, HPV DNA can be transferred via exosomes to stromal cells adjacent to tumor cells. The oncogenic effects of HPV are achieved by inhibiting the expression of p53, p21, and Rb suppressor genes in the tumor and adjacent tissues, as well as M2 macrophages polarization [95]. In the saliva of HPV-associated oral cancer patients, Wang et al. [96] isolated exosomes and successfully detected HPV16 DNA in salivary exosomes, having 80% consistency with HPV-16-positive oral cancer tissues. Therefore, HPV DNA in salivary exosomes can serve as a biomarker to assess the risk of HPV-associated oral cancer. Figure 3 summarizes virus-related components and roles in exosomes derived from HPV-associated cancers.

### 4.3. Hepatitis Virus-Related Cancers

The hepatitis virus is a general term for hepatitis A virus (HAV), HBV, HCV, hepatitis D virus (HDV), and hepatitis E virus (HEV), all named for their ability to cause hepatitis in humans. Among them, HAV and HEV are transmitted through intestinal infection [97], and the others through blood, sexual, or vertical transmission [98,99,100]. Deaths by viral hepatitis are mostly related to HBV and HCV [101]. In addition to chronic and alcoholic liver disease, intake of food contaminated with aflatoxins and cirrhosis by acute or chronic infection with HBV and HCV are involved in the development of hepatocellular carcinoma (HCC) [102]. HDV relies on HBV for virus particle assembly and dissemination. Similarly, HBV co-infection with HDV is more destructive [100,103]. The direct carcinogenic effect of HDV is controversial, but it can promote the oncogenic role of HBV by activating NF-κB and STAT3-mediated inflammatory responses and oxidative stress [104].

HBV can release many EVs, among which exosomes can affect HBV immunity [105,106]. Liu et al. [107] found that HBV-positive liver cancer cells were more resistant to chemotherapeutic drugs than HBV-negative liver cancer cells; the tumor volume decreased less after chemotherapy. HBV-related exosomes can activate chaperone-mediated autophagy and reduce HCC cell apoptosis, thereby inducing chemoresistance in HBV-related HCC. Yang et al. [108] identified an HBV-encoded miRNA, HBV-miR-3, highly expressed in HBV-positive liver cancer cells. In HBV-infected patients, HBV-miR-3 can be released via exosomes and HBV virions, inhibiting HBV protein and HBV replication. Zhao et al. [109] found that in HBV-infected HepG2-NTCP cells, HBV-miR-3 can be released into the extracellular space by exosomes, downregulate SOCS5, and activate the JAK/STAT pathway in HCC cells, thereby inhibiting HBV replication. They also found that HBV-miR-3 promoted polarization of M1 macrophages after downregulating SOCS5 and activating the STAT1 signaling pathway. Furthermore, HBV-miR-3 can downregulate PTEN and PPMIA protein expression, enhance HCC cell proliferation and invasion, and promote the development of HBV-related HCC [110,111]. A recent study found that HBV-miR-3 is only present in peripheral blood exosomes of chronic hepatitis B (CHB) patients, so it could be used for monitoring of HBV infection [112]. Liu et al. [113] found significantly lower miR-125b expression in serum exosomes of HCC patients than in patients with CHB and liver cirrhosis; low expression of exosomal miR-125b was associated with tumor number and tumor node metastasis staging. In clinical cases, HCC patients with low miR-125b expression had shorter time to recurrence and overall survival, maybe because exosomal miR-125b can inhibit HCC invasion and migration. Therefore, exosomal miR-125b may serve as a prognostic marker for recurrence and survival in HCC patients. Other studies have shown that more exosomes are secreted in HBx-expressing cells, and that HBx transcription and translation products in exosomes can be internalized into recipient cells, improving the microenvironment and promoting HBV spread [114]. According to the review of Ali et al. [115], HBx can affect growth, apoptosis, proliferation, migration and other biological functions of HCC cells by regulating miRNA expression, and affecting the occurrence and development of HCC.

**Figure 4:**
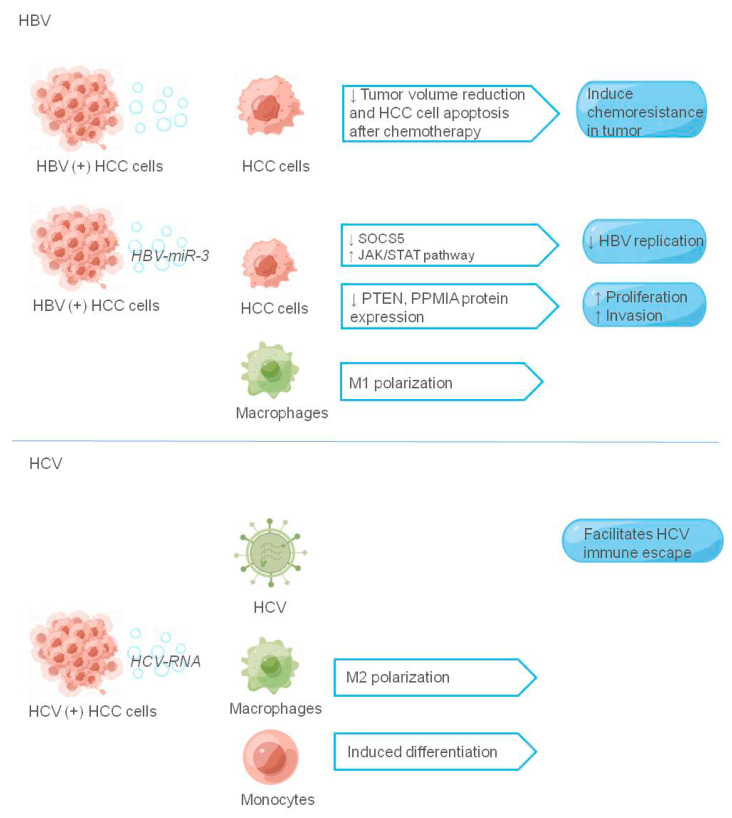
The role of exosomes in hepatitis virus-associated tumor microenvironment. The figure shows the liver cancer-related virus components and their receptors that play a role in exosomes, and the liver cancer-related virus components are shown in italics, ↑ indicating promotion and ↓ indicating inhibition.

In addition to HBV, HCV infection also plays a key role in the occurrence and development of HCC. Ramakrishnaiah et al. [116] detected the expression of viral protein and RNA in exosomes secreted by HCV-infected HCC Huh7.5.1 cells, and found that HCV could be transmitted through exosomes in HCC cells, leading to immune escape of the virus. Saha et al. [117] found that exosomes carrying HCV single-stranded RNA could induce monocyte differentiation and polarization of M2 macrophages through TLR7/8 in the same model. The exosome marker protein CD81 can bind to HCV envelope glycoprotein E2 and induce its maturation and secretion through exosomes. The HCV-CD81 complex is secreted out of the cell as exosomes infecting cells by membrane fusion [36]. Malik et al. [118] also found the immunomodulator CD81 in exosomes of HCC patients; CD81^+^ exosomes carried HCV particles, and this combination established persistent infection through immune escape mechanism, thus leading to HCC progression. Therefore, CD81^+^ exosomes may be a potential prognostic marker and therapeutic target for HCC caused by HCV infection. Figure 4 shows the role of hepatitis virus-associated tumor exosomes in the tumor microenvironment.

### 4.4. HIV-Related Cancers

Human immunodeficiency virus (HIV) is a retrovirus that causes chronic persistent infection in both humans and animals [119]. After HIV infection, the immune system is strongly attacked, especially CD4^+^T lymphocytes, the main target of HIV infection. In addition, in the final stage of HIV infection, that is, after entering the AIDS stage, the risk of developing malignant tumors is much higher than that of normal people; eventually, death is mostly due to secondary infection and/or cancer [120]. Three infection-related cancers are more common in HIV-infected individuals: Kaposi’s sarcoma associated with human herpetic virus 8 (HHV-8), invasive cervical cancer associated with HPV, and Hodgkin’s lymphoma associated with EBV [120,121].

It has long been shown that the transactivation response (TAR) element RNA acts as binding site of the HIV viral protein Tat and is the most abundant in HIV-1-derived miRNAs. Both HIV-infected primary cell exosomes and humanized mouse serum are enriched in TAR RNA, and TAR RNA-containing exosomes increase the susceptibility of undifferentiated naive cells to HIV-1 infection [122,123,124]. HIV TAR RNA not only enhances HIV susceptibility, but also promotes the growth and survival of cancer cells [125]. Chen et al. [126] found that in a patient infected with HIV, infected T cells could release exosomes to stimulate proliferation, invasion, and migration of oropharyngeal and lung cancer cells. Among them, HIV TAR RNA promotes cancer cell proliferation and induces the expression of proto-oncogenes and Toll-like receptor 3 (TLR3)-inducible genes. It can also rapidly enter recipient cells via EGFR, activating the EGFR/TLR3/ERK cascade, thereby promoting the growth and progression of cancer cells. In addition, exosomes purified from the saliva of HIV-positive patients or secreted by HIV-infected T cells promoted immortalization and Kaposi’s sarcoma herpesvirus (KSHV) infectivity in primary oral epithelial cells. The salivary exosomes of HIV-positive individuals contain TAR RNA, which can interact with EGFR and activate P38 MAPK signals, enhancing KSHV infectivity in oral epithelial cells; inhibiting EGFR may prevent KSHV infection and oral transmission [127].

**Figure 5:**
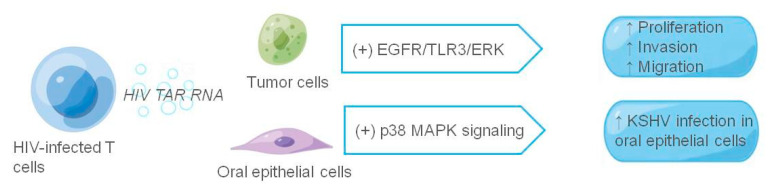
The role of exosomes in HIV-associated tumor microenvironment. The figure shows HIV-related components and their receptor cells at work in exosomes; HIV-related components are shown in italics, and ↑ indicates promotion.

In addition, HIV is inextricably linked with HPV and cervical cancer in women. Several studies have shown that the infection rate of HPV in women infected with HIV is higher than that of women not infected; continued HPV infection leads to an increase in the probability of cervical epithelial lesions. At the same time, HPV infection is closely related to cervical cancer; therefore, HIV infection will also increase the prevalence of cervical cancer in women [128,129,130,131,132]. Li et al. [133] found that exosomal miR-155-5p secreted by HIV-infected T cells could downregulate ARID2, a direct target gene in cervical cancer cells, to activate the ERCC5-NF-κB signaling pathway, promoting the proliferation, migration, and invasion of cervical cancer cells. The study also found that miR-155-5p could induce IL-6 and IL-8 secretion, thus promoting cancer cell proliferation, stemness and tumorigenicity, and survival. This article summarizes the relationship between exosomes released from HIV-infected cells and the tumor microenvironment (Figure 5).

## 5. Conclusions

Exosomes have been attracting the attention of researchers since they were first discovered in 1983, and the understanding of exosomes has developed at an astonishing rate. At the beginning, exosomes were considered as a way for cells to excrete waste. Later, with many studies on their biological origin, substance composition, occurrence process, signal transduction, and distribution in body fluids, various functions of exosomes have been gradually enriched. The occurrence of tumors is not only determined by the tumor tissue and cells themselves, but also closely related to the microenvironment in which the tumor is located. Tumor and environment are interdependent, mutually promoting, and mutually antagonistic. Exosomes, having a crucial role in cell-to-cell communication, are also involved in cancer. The function of exosomes depends on the cell type from which derive, and they are involved in the body’s immune response, antigen presentation, cell migration, cell differentiation, and tumor invasion. Studying the relationship between exosomes and the tumor microenvironment is not only of great significance for understanding tumor occurrence, development, and metastasis, but also plays an important role in tumor diagnosis, prevention and treatment, and prognosis. This review focuses on describing the role of exosomes in the virus-associated tumor microenvironment and provides a summary (Table 1).

## Figures and Tables

**Table 1:** Effects of exosomes secreted by virus-associated tumor cells on the tumor microenvironment. The components and their functions in virus-related tumor exosomes mentioned in the article are listed in the table.

Cancers	Components in Exosomes		Function	References
EBV-related Cancers	HIF-1α		Promote the migration and invasion of NPC cells	[64]
	LMP1		Promotes migration and invasion of EBV-associated tumor cells Involved in immune regulation	[65,66]
	galectin-9		Involved in immune regulation, inducing apoptosis of Th1 lymphocytes	[67]
	miR-BART-10-5p		Promote angiogenesis in nasopharyngeal carcinoma	[71]
	miR-18a			
	EBERs		Trigger antiviral immunity	[74]
			Induction of malignant transformation of EBV-negative cell lines	[75,76]
HPV-related cancers	HPV E6/E7		Induces CD8+ T cell immunity and inhibits tumor growth	[86]
			Anti-tumor immune function in head and neck cancer	[87]
	HPV DNA		Activates breast cancer stromal cells and promotes breast cancer cell proliferation and invasion	[94]
			Polarize macrophages into M2 type and play a carcinogenic role	[95]
Hepatitis Virus-associated Cancers	HBV	HBV-associated exosomes	Affect HBV immunity	[105,106]
			Induction of chemoresistance in hepatocellular carcinoma	[107]
		HBV-miR-3	Inhibition of HBV replication in hepatocellular carcinoma and promotion of M1 macrophage polarization	[109]
			Enhanced hepatocellular carcinoma proliferation and invasion	[110,111]
	HCV	HCV-RNA	Facilitates immune escape of HCV in liver cancer cells	[116]
			Induction of monocyte differentiation and M2 macrophage polarization	[117]
HIV-related Cancers	TAR RNA		Promote tumor cell proliferation	[126]
			Promotes KSHV infection of oral epithelial cells	[127]
	miR-155-5p		Promote tumor cell proliferation, stemness and tumorigenicity	[133]
